# A prospective multicentre screening study on multidrug-resistant organisms in intensive care units in the Dutch–German cross-border region, 2017 to 2018: the importance of healthcare structures

**DOI:** 10.2807/1560-7917.ES.2022.27.5.2001660

**Published:** 2022-02-03

**Authors:** Corinna Glasner, Matthijs S Berends, Karsten Becker, Jutta Esser, Jens Gieffers, Annette Jurke, Greetje Kampinga, Stefanie Kampmeier, Rob Klont, Robin Köck, Lutz von Müller, Nashwan al Naemi, Alewijn Ott, Gijs Ruijs, Katja Saris, Adriana Tami, Andreas Voss, Karola Waar, Jan van Zeijl, Alex W Friedrich

**Affiliations:** 1 Department of Medical Microbiology and Infection Control, University of Groningen, University Medical Center Groningen, Groningen, the Netherlands; 2 Certe Medical Diagnostics and Advice Foundation, Groningen, the Netherlands; 3 Institute of Medical Microbiology, University Hospital Münster, Münster, Germany; 4 Friedrich Loeffler-Institute of Medical Microbiology, University Medicine Greifswald, Greifswald, Germany; 5 Practice of Laboratory Medicine and University Osnabrück, Department of Dermatology, Environmental Medicine and Health Theory, Osnabrück, Germany; 6 Institute for Microbiology, Hygiene and Laboratory Medicine, Klinikum Lippe, Detmold, Germany; 7 North Rhine-Westphalian Centre for Health, Section Infectious Disease Epidemiology, Bochum, Germany; 8 Institute of Hygiene, University Hospital Münster, Münster, Germany; 9 Laboratory Microbiology Twente Achterhoek, Hengelo, the Netherlands; 10 Institute of Hygiene, DRK Kliniken Berlin, Berlin, Germany; 11 Institute for Laboratory Medicine, Microbiology and Hygiene, Christophorus-Kliniken GmbH, Coesfeld, Germany; 12 Laboratory for Medical Microbiology and Infectious Diseases, Isala, Zwolle, the Netherlands; 13 Department of Medical Microbiology, Radboud University Medical Centre and Canisius-Wilhelmina Hospital, Nijmegen, the Netherlands; 14 Izore, Centre for Infectious Diseases Friesland, Leeuwarden, the Netherlands; 15 European Prevention Networks in Infection Control, University Hospital Münster, Münster, Germany

**Keywords:** multidrug-resistant organisms, MDRO, bacteria, European cross-border region, intensive care unit, screening, healthcare structures

## Abstract

**Background:**

Antimicrobial resistance poses a risk for healthcare, both in the community and hospitals. The spread of multidrug-resistant organisms (MDROs) occurs mostly on a local and regional level, following movement of patients, but also occurs across national borders.

**Aim:**

The aim of this observational study was to determine the prevalence of MDROs in a European cross-border region to understand differences and improve infection prevention based on real-time routine data and workflows.

**Methods:**

Between September 2017 and June 2018, 23 hospitals in the Dutch (NL)–German (DE) cross-border region (BR) participated in the study. During 8 consecutive weeks, patients were screened upon admission to intensive care units (ICUs) for nasal carriage of meticillin-resistant *Staphylococcus aureus* (MRSA) and rectal carriage of vancomycin-resistant *Enterococcus faecium*/*E. faecalis* (VRE), third-generation cephalosporin-resistant Enterobacteriaceae (3GCRE) and carbapenem-resistant Enterobacteriaceae (CRE). All samples were processed in the associated laboratories.

**Results:**

A total of 3,365 patients were screened (median age: 68 years (IQR: 57–77); male/female ratio: 59.7/40.3; NL-BR: n = 1,202; DE-BR: n = 2,163). Median screening compliance was 60.4% (NL-BR: 56.9%; DE-BR: 62.9%). MDRO prevalence was higher in DE-BR than in NL-BR, namely 1.7% vs 0.6% for MRSA (p = 0.006), 2.7% vs 0.1% for VRE (p < 0.001) and 6.6% vs 3.6% for 3GCRE (p < 0.001), whereas CRE prevalence was comparable (0.2% in DE-BR vs 0.0% in NL-BR ICUs).

**Conclusions:**

This first prospective multicentre screening study in a European cross-border region shows high heterogenicity in MDRO carriage prevalence in NL-BR and DE-BR ICUs. This indicates that the prevalence is probably influenced by the different healthcare structures.

## Introduction

Antimicrobial resistance (AMR) is a growing public health threat worldwide. Specifically, multidrug-resistant organisms (MDRO) pose major health risks to humans both in the community and within healthcare facilities [1,2]. Hospitals are particularly exposed to this risk and are challenged at multiple levels, e.g. the individual patient, the healthcare team, the organisation and the political and economic environment. In hospitals, patients colonised and/or infected with MDROs have prolonged hospital stays, higher risks for complications, and an increased morbidity and mortality, all of which increase healthcare costs [3,4]. To decrease these risks, the World Health Organization (WHO) has urgently advised changing the way antibiotics are prescribed. In addition, the WHO highlighted that behavioural changes, resulting from the implementation of infection prevention measures, are indispensable to successfully combat AMR [5,6]. According to WHO’s analyses, one key pitfall is that international AMR surveillance is neither coordinated nor harmonised. Currently, there are still information gaps, especially with respect to twelve MDROs, which have been categorised as urgently requiring new antibiotics and improved combat strategies [6,7]. These MDROs include among others: meticillin-resistant *Staphylococcus aureus* (MRSA), vancomycin-resistant *Enterococcus faecium* (VRE), extended spectrum beta-lactamase (ESBL)-producing Enterobacteriaceae and carbapenem-resistant Enterobacteriaceae (CRE) [7]. The prevalence of such MDROs varies not only between countries, but also between different regions (henceforth called ‘healthcare regions’), within one country or areas that comprise cross-border regions, such as the Dutch–German cross-border region [8,9].

Hospital transfer of patients within or between healthcare regions, i.e. from a local or regional hospital to a university medical centre or vice versa, can be a substantial driver of AMR [9]. Thus, prevalence estimates of MDROs at the regional level may better reflect the actual reality and allow the implementation of interventions more effectively. This knowledge is of utmost importance, especially since the European Union (EU) directive from 2011 allows patients to seek medical treatment in any EU country. As ca 30% of all EU citizens live in a cross-border region, this underlines the importance of a non-national-only, but a regional cross-border approach.

The Dutch–German cross-border region has been at the forefront of cooperation in the domain of AMR and infection prevention since 2005 with the support of European INTERREG programmes (www.deutschland-nederland.eu). Since then, the projects developed within the INTERREG programme have been denoted a ‘best practice’ for studying the prevalence of MDRO in a European cross-border region (Interact, European Cooperation Day, 2013). Importantly, among all cross-border regions in Europe, the Dutch–German cross-border region exhibits the most frequent exchange of citizens, with 74% of Germans and Dutch citizens living close to the border indicating to have visited the other country [10]. Additionally, patient movements, e.g. exchange of patients between different healthcare institutes, across this particular border occur on a regular basis [9].

A recently published comparison of the national Dutch and German guidelines on Gram-negative MDROs urged the usage of consistent terminology and harmonised diagnostic procedures for the improvement of infection prevention, treatment and patient safety [11]. Gathering and comparing regional data from both sides of the border was considered essential because of two reasons. Firstly, the directive 2011/24/EU [12] will lead to an increasing number of patients seeking medical treatment in a neighbouring country. Secondly, the number of neonates, as well as immuno-compromised and elderly patients who are seeking treatment will also continue to increase particularly in cross-border regions between two high-income countries with cost-intensive, highly advanced and technologically driven healthcare systems [13].

With the advancements in healthcare, demographic changes and an increase in the number of multimorbidities, intensive care units (ICUs) have become the central point in many in-hospital patient flows [14,15]. ICUs represent a distinct hospital environment with frequent contact between specially trained hospital staff and critically ill patients who require advanced technology and increased antibiotic prescription [16]. Thus, ICUs are catalysing the emergence and transmission of MDROs, frequently causing infections in critically ill patients [17].

Therefore, the aim of this observational prospective multicentre screening study was to determine the prevalence of selected MDROs on admission to adult ICUs in the Dutch–German cross-border region (NL-DE-BR). The analyses are based on real-time routine data and workflows in order to correlate those with the existing healthcare structures.

## Methods

### Study design and setting

This observational prospective multicentre screening study was conducted between 1 September 2017 and 18 June 2018 in the NL-DE-BR to determine the prevalence of MDROs in adult ICUs. All adult patients (aged ≥ 18 years) were included in the study. The screening period for all hospitals lasted 8 consecutive weeks (Supplementary Figure S1).

A total of 23 hospitals, eight Dutch and 15 German, participated in this study. The 23 hospitals were served by 10 laboratories, six on the Dutch side (Dutch border region (NL-BR)) and four on the German side (German border region (DE-BR)). Both regions, which together comprise the NL-DE-BR, have a similar geographical size, population density and type of hospital care (one university hospital, and several secondary care hospitals (non-university hospitals)). Data about the number of beds per hospital and ICU, hospital and ICU admissions and hospital and ICU patient days were provided by all participating hospitals for 2016.

During the screening period, each participating hospital aimed to screen all patients at admission to their ICU for nasal carriage of MRSA and rectal carriage of VRE (both *E. faecium* and *E. faecalis*), both Gram-positive pathogens, and third-generation cephalosporin-resistant Enterobacteriaceae (3GCRE) and CRE, both Gram-negative pathogens. For the definition of 3GCRE, the European Centre for Disease Prevention and Control (ECDC) guideline was followed; all three antibiotics, cefotaxime, ceftazidime and ceftriaxone, were considered for the definition of 3GCRE. Moreover, although defined as Enterobacteriaceae, the present study focussed solely on *Escherichia coli* and *Klebsiella* spp. An overview of all MDRO definitions used in this study is summarised in the Supplementary Material.

### Laboratory investigations

All samples were processed at the associated routine diagnostic laboratory, which were all International Organisation for Standardization (ISO) certified at the time of the study. All laboratories followed local standard operating procedures, which were adapted to the study protocol when necessary (Supplementary Table S1). Bacterial species were confirmed by MALDI-TOF mass spectrometry and antibiotic susceptibility was determined using VITEK 2 automated systems (bioMérieux, Inc, Durham, North Carolina, United States) with the usage of the European Committee on Antimicrobial Susceptibility Testing (EUCAST) clinical breakpoints [18]. 

### Statistical analysis and software

Data analysis was done in R using the software application RStudio and the R package “AMR” (R v4.0.2, RStudio v1.3.959 and AMR package v1.3.0; R Foundation, Vienna, Austria), which are all cost-free, open-source and publicly available [19].

Contingency tables were tested with Fisher’s exact test when the size was 2 x 2 and chi^-^squared tests otherwise. To test for equality in prevalence between countries, the exact binomial test was used. Outcomes of statistical tests were considered significant when two-sided p < 0.05.

### Ethical statement

The medical ethical committee of the University Medical Center Groningen (UMCG, the Netherlands) was informed and patients or their relatives were approached to voluntarily participate in the study. Ethical approval and informed consent were not required (METc 2015.535). All data were collected in accordance with the European Parliament and Council decisions on the epidemiological surveillance and control of communicable disease in the European Community [20]. The board of directors of all other participating hospitals agreed to conduct the study.

## Results

Between 1 September 2017 and 18 June 2018, 23 hospitals in the NL-DE-BR participated in the study, eight in the NL-BR and 15 in the DE-BR. The total number of beds from all participating ICUs was 443 (NL-BR: n = 182 (41.1%); DE-BR: n = 261 (58.9%)). The bed capacity of the ICUs in relation to the respective hospital bed capacity did not differ between hospitals within either country or between the two countries (NL-BR: 3.2% (IQR: 3.0–3.7); DE-BR: 3.6% (IQR: 1.8–5.5)). The participating hospitals are characterised by the data shown in Table 1.

**Table 1:** Overview of hospital and intensive care unit capacity, Dutch–German cross-border region, 2016

Parameters	Border region						p value
	NL-BR		DE-BR		NL-DE-BR		
Hospitals (n)	8		15		23		NA
Laboratories (n)	6		4		10		NA
Beds	n	IQR	n	IQR	n	IQR	p value
Hospital, total	7,514	NA	5,388	NA	12,902	NA	NA
Hospital, median per hospital	591	416–900	436	266–620	476	330–683	0.43
ICU, total	182	NA	261	NA	443	NA	NA
ICU, median per hospital	19.0	13.5–32.0	14	10.0–22.0	15.5	12.0–22.0	0.51
ICU beds of all local beds (%)	3.2	3.0–3.7	3.6	1.8–5.5	3.3	2.9–4.7	0.37
Admissions							
Hospital	29,872	27,261–34,265	22,009	11,332–30,851	25,498	14,698–31,661	0.20
ICU	1,277	854–2,726	1,307	895–1,932	1,307	895–1,993	0.77
ICU per 100 hospital admissions	4.8	3.5–7.0	7.7	4.2–10.5	6.8	4.1–9.2	0.08
Patient days							
Hospital	146,515	135,774–179,734	116,866	79,882–182,395	137,512	102,407–182,395	0.80
ICU	5,395	3,853–9,650	4,596	3,038–7,288	4,707	3,346–7,288	0.69
Average length of stay in days							
Hospital	4.98	4.46–5.23	6.10	5.80–6.71	5.83	5.09–6.54	0.03
ICU	4.06	3.53–4.26	3.57	2.77–3.81	3.71	3.10–4.18	0.84

DE-BR: German border region; ICU: intensive care unit; IQR: interquartile range; NA: not applicable; NL-BR: Dutch border region; NL-DE-BR: Dutch–German cross-border region.

Hospital and ICU admissions, patient days and average length of stay in days are median values.

### Study population and screening samples from intensive care units 

A total of 3,365 patients were screened: 1,202 (35.7%) in NL-BR ICUs and 2,163 (64.3%) in DE-BR ICUs (Table 2). The screening period per hospital lasted 8 consecutive weeks (n = 56 days; IQR: 55–58) (Supplementary Figure S1). In both NL-BR and DE-BR, significantly more men than women were screened (p < 0.001) and in NL-BR fewer women were screened than in DE-BR (p < 0.01). The median age of all screened patients was 68 years (IQR: 57–77), while patients in DE-BR were significantly older than patients in the NL-BR (p < 0.001).

**Table 2:** Overview of the total number of patients present (n = 5,568) and screened (n = 3,365), swabs and type of bacteria tested for in the Dutch–German cross-border region, September 2017–June 2018

Screening for MDROs	NL-BR	DE-BR	NL-DE-BR	p value
Overall				
Patients present (%)	2,111 (37.9)	3,457 (62.1)	5,568	NA
Patients screened (%)	1,202 (35.7)	2,163 (64.3)^a^	3,365	NA
Overall screening compliance, %^b^	56.9	62.6	60.4	< 0.001
Men screened (%)	757 (63.0)	1,253 (57.9)	2,010 (59.7)	0.004
Women screened (%)	445 (37.0)	910 (42.1)	1,355 (40.3)	0.004
Median age of patients screened, years (IQR)	66 (55–73)	69 (58–79)	68 (57–77)	< 0.001
Swabs taken	2,308	4,154	6,462	NA
MRSA				
Patients screened	1,174	2,117	3,291	NA
Positive patients (prevalence, %)	7 (0.6)	36 (1.7)	43 (1.3)	0.006
Positive ICU patients/100 hospital admissions^c^	0.02	0.07	0.05	NA
Positive patients/100 ICU admissions^c^	0.33	1.04	0.77	NA
VRE				
Patients screened	1,110	2,035	3,145	NA
Positive patients (prevalence, %)	1 (0.1)	55 (2.7)	56 (1.8)	< 0.001
Positive ICU patients/100 hospital admissions^c^	0.003	0.11	0.06	NA
Positive patients/100 ICU admissions^c^	0.05	1.59	1.00	NA
3GCRE				
Patients screened	1,126	2,026	3,152	NA
Positive patients (prevalence, %)	40 (3.6)	133 (6.6)	173 (5.5)	< 0.001
Positive ICU patients/100 hospital admissions^c^	0.10	0.26	0.19	NA
Positive patients/100 ICU admissions^c^	1.86	3.85	3.09	NA
CRE				
Patients screened	1,126	2,026	3,152	NA
Positive patients (prevalence, %)	0 (0)	4 (0.2)	4 (0.1)	0.30
Positive ICU patients/100 hospital admissions^c^	0	0.008	0.005	NA
Positive patients/100 ICU admissions^c^	0	0.11	0.07	NA

3GCRE: third-generation cephalosporin-resistant Enterobacteriaceae; CRE: carbapenem-resistant Enterobacteriaceae; DE-BR: German border region; ICU: intensive care unit; IQR: interquartile range; MDRO: multidrug resistant organism; MRSA: meticillin-resistant *Staphylococcus aureus*; NA: not applicable; NL-BR: Dutch border region; NL-DE-BR: Dutch–German cross-border region; VRE: vancomycin-resistant enterococci.

^a^ Missing sex information for three patients from DE-BR.

^b^ Screening compliance was defined as percentage of patients screened for at least one MDRO group.

^c^ Observed positive patients were extrapolated for years, i.e. the results of the 8 consecutive screening weeks were multiplied by 6.5 to a total of 52 weeks to be able to normalise by the year’s total of number of admissions.

A total of 6,462 swabs were taken, 2,308 (35.7%) in NL-BR and 4,154 (64.3%) in DE-BR ICUs (Table 2). Of those, 3,292 (51%) were taken from the nasopharynx and 3,170 (49%) were from the rectum. The overall screening compliance (screened for at least one MDRO group) was 60.4% (3,365/5,568 patients). For ICUs in the NL-BR this was 56.9% (1,202/2,111) and for ICUs in the DE-BR this was 62.6% (2,163/3,457), p < 0.001. The median screening compliance for all four MDRO groups (i.e. nasopharyngeal swab for MRSA, rectal swab for VRE, 3GCRE and CRE) on the other hand was in total 55.3% (3,081/5,568), and 52.1% (1,100/2,111) in NL-BR and 57.3% (1,981/3,457) in DE-BR ICUs (p < 0.001). Most patients (91.5% for NL-DE-BR ICUs) that were screened while present in the ICU were screened for all MDRO groups.

In total, 3,291 patients were screened for MRSA (n = 1,174; 35.7% in NL-BR and n = 2,117; 64.3% in DE-BR ICUs), 3,145 for VRE (n = 1,110; 35.3% in NL-BR and n = 2,035; 64.7% in DE-BR ICUs) and 3,152 for 3GCRE (n = 1,126; 35.7% in NL-BR and n = 2,026; 64.3% in DE-BR ICUs). Of note, in some patients, multiple MDROs were found from the same or different species, meaning that some patients are included in multiple MDRO groups.

### Prevalence of Gram-positive MDROs: MRSA and VRE

**Figure 1:**
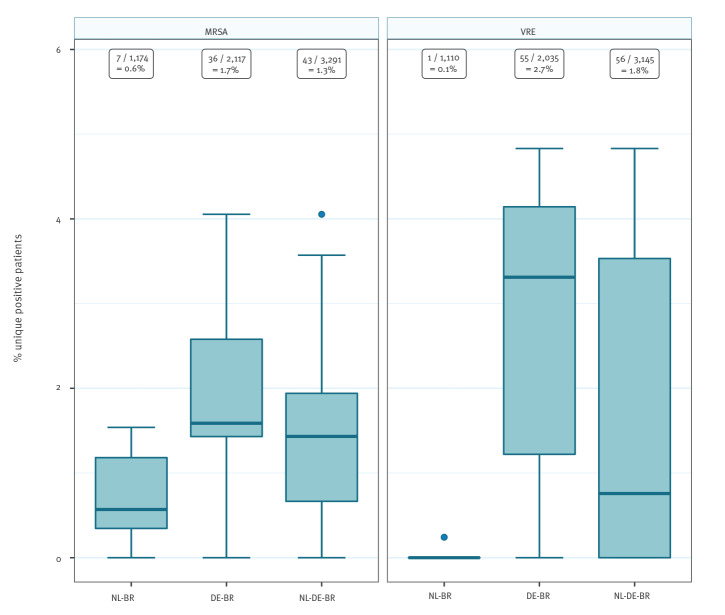
Prevalence of MRSA (n = 3,219) and VRE (n = 3,145) in intensive care units in the Dutch–German cross-border region, September 2017–June 2018

DE-BR: German border region; ICU: intensive care unit; IQR: interquartile range; MRSA: meticillin-resistant *Staphylococcus aureus*; NL-BR: Dutch border region; NL-DE-BR: total Dutch–German cross-border region; VRE: vancomycin-resistant enterococci.

The overall prevalence for MRSA carriage at ICU admission was 1.3% (43/3,291), and VRE carriage was 1.8% (56/3,145). The prevalence was higher in DE-BR than in NL-BR ICUs, namely 1.7% (36/2,117) vs 0.6% (7/1,174) for MRSA (p = 0.006) and 2.7% (55/2,035) vs 0.1% (1/1,110) for VRE (p < 0.001), respectively (Figure 1). The prevalence ranged from 0% to 1.5% in NL-BR ICUs and from 0% to 4.1% in DE-BR ICUs for MRSA and from 0% to 0.3% in NL-BR ICUs and from 0% to 4.8% in DE-BR ICUs for VRE (Figure 1). An overview of all isolated MRSA and VRE isolates can be found in the Supplementary Table S2. Notably, all 56 cases of VRE were caused by *E. faecium*.

The square boxes represent the number of positive patients divided by the total number of patients screened for the respective pathogen with the calculated prevalence. Boxplots show the median prevalence in participating ICUs (thick line within each box), the first and third quartile (the difference of the upper and lower borders of the box represents the IQR), and the whiskers with error bars represent 1.5 times the IQR, denoting the normal range. The dots represent data points outside this range.

### Prevalence of Gram-negative MDROs: 3GCRE and CRE

**Figure 2:**
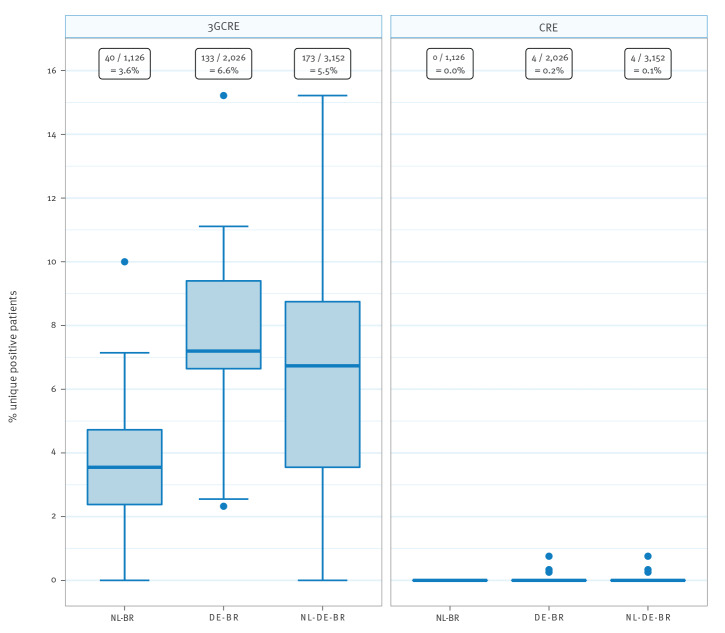
Prevalence of 3GCRE and CRE in intensive care units in the Dutch–German cross-border region, September 2017–June 2018 (n = 3,152)

DE-BR: German border region; ICU: intensive care unit; IQR: interquartile range; MRSA: meticillin-resistant *Staphylococcus aureus*; NL-BR: Dutch border region; NL-DE-BR: total Dutch–German cross-border region; VRE: vancomycin-resistant enterococci.

The overall prevalence at ICU admission for 3GCRE carriage was 5.5% (173/3,152) and 0.1% (4/3,152) for CRE carriage. The prevalence for 3GCRE was significantly higher in DE-BR than in NL-BR ICUs, namely 6.6% (133/2,026) vs 3.6% (40/1,126; p < 0.001), whereas the prevalence for CRE was comparable, with 0.0% (0/1,126) in NL-BR ICUs vs 0.2% (4/2,026) in DE-BR ICUs (Figure 2 and Table 2). Most of the isolated 3GCRE were *E. coli* isolates (166/3152; 92.2%). Twelve isolates were *K. pneumoniae* (6.8%), one *K. variicola* (0.6%) and one *K. oxytoca* (0.6%). The four CRE isolates were found in three different DE-BR ICUs, three were *E. coli* and one was a *K. pneumoniae* isolate. The prevalence for 3GCRE differed within both countries between hospitals, ranging from 0% to 10.0% in NL-BR ICUs and from 2.3% to 15.2% in DE-BR ICUs (Figure 2). Table 2 presents an overview of the prevalence of MRSA, VRE, 3GCRE and CRE. An overview of all isolated 3GCRE and CRE isolates can be found in the Supplementary Table S2.

The square boxes represent the number of positive patients divided by the total number of patients screened for the respective pathogen with the calculated prevalence. Boxplots show the median prevalence in participating ICUs (thick line within each box), the first and third quartile (the difference of the upper and lower borders of the box represents the IQR), and the whiskers with error bars represent 1.5 times the IQR, denoting the normal range. The dots represent data points outside this range.

### Prevalence of Gram-negative MDROs based on Dutch and German definitions

The national guidelines for the Netherlands and Germany differ greatly in the way Gram-negative MDROs are defined, while definitions for MRSA and VRE are identical [13,21]. An overview of the specific Dutch and German definitions of MDROs is summarised in the Supplementary Material.

**Figure 3:**
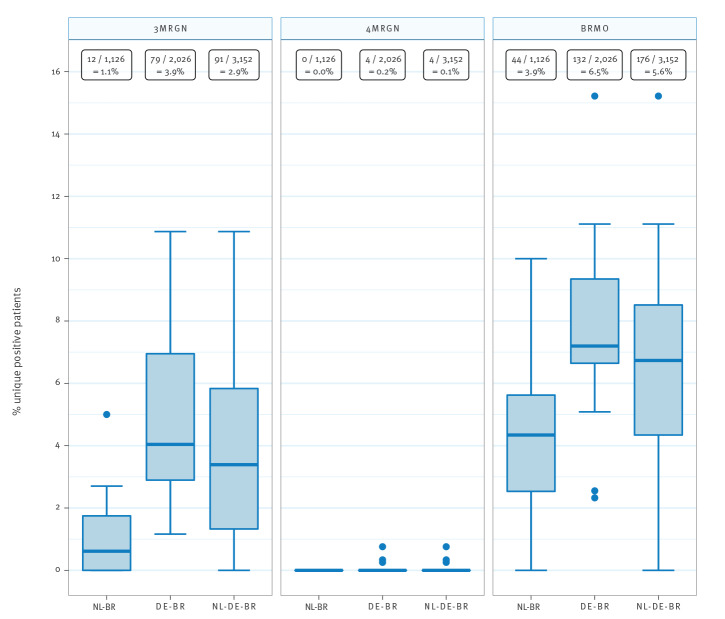
Prevalence of 3MRGN, 4MRGN and BRMO in intensive care units in the Dutch–German cross-border region, September 2017–June 2018 (n = 3,152)

The German national infection prevention guideline classifies Gram-negative MDROs into 3MRGN and 4MRGN (German: ‘Multiresistente Gram-negative Stäbchen’, multidrug-resistant Gram-negative rods) based on phenotypic susceptibility. When the German MRGN definition is applied to all Gram-negative isolates, the overall prevalence for 3MRGN is 2.9% (91/3,152) and for 4MRGN 0.1% (4/3,152). The prevalence was significantly lower in NL-BR than in DE-BR ICUs for 3MRGN, namely 1.1% (12/1,126) vs 3.9% (79/2,026) (p < 0.001), whereas the prevalence for 4MRGN was comparable, namely 0% (0/1,126) vs 0.2% (4/2,026) (p = 0.30) (Figure 3). The prevalence for 3MRGN differed within both countries between hospitals, ranging from 0% to 5.0% in NL-BR and from 1.2% to 10.9% in DE-BR ICUs. The four 4MRGN were three *E. coli* isolates and one *K. pneumoniae* isolate and originated from three different DE-BR ICUs. Of note, for the definition of 3MRGN, piperacillin results could not be included, since only results for piperacillin-tazobactam were reported.

3MRGN: Multiresistente Gram-negative Stäbchen mit Resistenz gegen 3 der 4 Antibiotikagruppen (multiresistant Gram-negative rods with resistance to 3 of the 4 antibiotic groups); 4MRGN: Multiresistente Gram-negative Stäbchen mit Resistenz gegen 4 der 4 Antibiotikagruppen (multidrug-resistant Gram-negative rods with resistance to 4 of the 4 antibiotic groups); BRMO: bijzonder-resistente microorganism (particularly resistant microorganisms); DE-BR: German border region; ICU: intensive care unit; IQR: interquartile range; NL-BR: Dutch border region; NL-DE-BR: Dutch–German cross-border region.

The square boxes represent the number of positive patients divided by the total number of patients screened for the respective pathogen with the calculated prevalence. Boxplots show the median prevalence in participating ICUs (thick line within each box), the first and third quartile (the difference of the upper and lower borders of the box represents the IQR), and the whiskers with error bars represent 1.5 times the IQR, denoting the normal range. The dots represent data points outside this range.

The Dutch national guideline defines exceptional resistant microorganisms as BRMO (‘Bijzonder Resistente Microorganismen’) using strict interpretation guidelines [22]. When the Dutch BRMO definition is applied to all Gram-negative isolates, the overall BRMO prevalence is 5.6% (176/3,152). The prevalence was lower in NL-BR than in DE-BR ICUs, namely 3.9% (44/1,126) vs 6.5% (132/2,026) for BRMOs (p = 0.002) (Figure 3). The prevalence for BRMO differed within both countries between hospitals, ranging from 0% to 10.0% in NL-BR and from 2.3% to 15.2% in DE-BR ICUs.

### Comparison of MDRO prevalence between NL-BR and DE-BR ICUs in university and non-university hospitals

**Figure 4:**
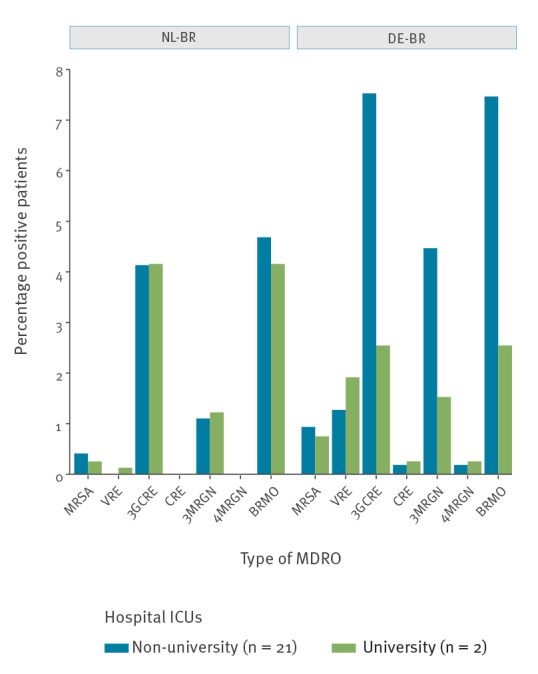
Comparison of multidrug-resistant organism prevalence between non-university and university hospital intensive care units in the Dutch–German cross-border region, September 2017–June 2018

For NL-BR ICUs, the prevalence of all MDRO groups was not significantly different between non-university hospitals (n = 7) and the university hospital (n = 1) (Figure 4). In participating DE-BR ICUs, the prevalence of 3GCRE (p < 0.001), 3MRGN (p = 0.005) and BRMO (p < 0.001) were significantly higher in the non-university hospitals (n = 22) (Figure 4). Interestingly, the prevalence of almost all investigated MDROs was not significantly different between the two university hospitals, except for the prevalence of VRE, which was significantly higher in the German university ICU (p < 0.001). Comparing the prevalence of all investigated MDROs between NL-BR and DE-BR non-university hospital ICUs revealed a significant difference for VRE (p < 0.001), 3GCRE (p < 0.001), 3MRGN (p < 0.001) and BRMO (p < 0.001), whereas the difference for MRSA (p = 0.83) differed only slightly (Figure 4).

3MRGN: multiresistant Gram-negative rods with resistance to 3 of the 4 antibiotic groups; 4MRGN: multidrug-resistant Gram-negative rods with resistance to 4 of the 4 antibiotic groups; 3GCR: third-generation cephalosporin-resistant Enterobacteriaceae; BRMO: particularly resistant microorganisms; DE-BR: German border region; ICU: intensive care unit; IQR: interquartile range; MRSA: meticillin-resistant *Staphylococcus aureus*; NL-BR: Dutch border region; NL-DE-BR: total Dutch–German cross-border region; VRE: vancomycin-resistant enterococci, CRE: carbapenem-resistant Enterobacteriaceae.

The NL-BR has one university hospital and seven non-university hospitals and the DE-BR has one university hospital and 14 non-university hospitals that participated in the study.

## Discussion

To the best of our knowledge, this is the first prospective observational multicentre screening study focussing on ICU admission prevalence of the most common MDROs in a healthcare region that comprises a national border. This study has been performed by a team within the Dutch–German cross-border network, which has a long-lasting experience in close cooperation in the domain of AMR and infection prevention and control [23,24]. Interestingly, the Dutch and German healthcare systems differ in many aspects, creating a natural ‘living lab’ situation to study AMR and other healthcare-related topics. One difference is the overall hospital activity. In the NL-BR, 4.8 per 100 hospital admissions lead to an ICU admission. In contrast, in the DE-BR, this number is 7.7 per 100 hospital admissions. This difference can be explained by the higher ICU capacity in DE-BR hospitals, namely 4.8% vs 2.4% in NL-BR. Interestingly, the median hospital-wide length of stay (LOS) is shorter in the NL-BR than in the DE-BR (4.98 vs 6.10 days), whereas the ICU-specific LOS is longer in the NL-BR (4.06 vs 3.57 days). When comparing our data with the LOS by Eurostat from 2017, it can be observed that the hospital-wide LOS of the NL-BR is comparable to the national average (5.0 vs 4.5 days), whereas for Germany, the LOS of the DE-BR is much lower (6.1 vs 9.0) [21]. Although no information was available for the present study with regard to staffing in hospitals and ICUs, it has been shown by others that the number of available staff in German ICUs is much less than in Dutch ICUs, while understaffing has been found to be inversely proportional to the detection of MDROs [16,25,26]. Strikingly, a more recent study focussing on the NL-DE-BR presented that healthcare workers on both sides of the border have a similar awareness and perception towards AMR and both struggle with the limitations to cope with the application of preventive measures [27].

The success of infection prevention and other actions to prevent and control AMR within a hospital can be measured by the occurrence of MDROs. To this end, the ECDC reports overviews of MDRO proportions based on nationally aggregated data from blood cultures on a regular basis. On a more country-specific level, MDRO proportions are also reported by national health institutes (NHI) – the Rijkinstituut voor Volgsgezondheid en Milieu (RIVM) in the Netherlands and the Robert-Koch Institute (RKI) in Germany [28,29]. These MDRO proportions differ greatly from the prevalence of MDRO carriage reported here. MDRO proportions are the fraction of e.g. MRSA isolates among *S. aureus* isolates, whereas MDRO prevalence is the fraction of patients with e.g. MRSA colonisation in a certain patient population. MDRO proportions are thus based on the microorganism and the respective resistance pattern, information that can be easily extracted from any laboratory information system, whereas MDRO prevalence is based on the patient or a certain population and requires mostly active screening. While both are of high importance and serve different purposes, only MDRO prevalence informs us about the carriage or infection rate in patients.

In the present study, the overall carriage prevalence for the different MDROs was higher in the DE-BR ICUs, although some differences were marginal. Specifically, prevalence of MRSA carriage was three times higher in the DE-BR (1.7%) than in the NL-BR (0.6%). These prevalences are consistent with a recent study about all nosocomial MRSA cases in this region from 2012 until 2016 [24]. For 2018, reports on the country level published by the ECDC show that the proportion of MRSA among *S. aureus* isolates from blood cultures was 1.2% in the Netherlands and 7.6% in Germany (with regional variations as per the Dutch and German NHIs, e.g. 0.3% in the Northern Netherlands and 14.5% in Northern-West Germany in any blood culture) [28-30]. Differences between proportions and prevalence are of course expected, and the higher MRSA proportions can, for example, be explained by an increased antibiotic use to foster the occurrence of MRSA. Nevertheless, the rather low prevalence of MRSA carriage on both sides of the border demonstrates that national efforts to control MRSA specifically in this cross-border region, which have been continuously successful in the Netherlands for decades, have now led to a decrease on the German side of the border as well.

For VRE, the prevalence measured in this study was 0.1% in the NL-BR and also remained low in the DE-BR (2.7%), although almost 30 times higher than in the NL-BR. This difference is also reflected by different proportions of VRE among *E. faecium* from blood: 1.1% in the Netherlands vs 23.8% in Germany in 2018 as reported by the ECDC, and 0.6% vs 7.6% in any blood culture in 2018 as reported by the Dutch and German NHIs, respectively [28-30]. The large difference in the German VRE proportion between the data from ECDC and the German NHI cannot be explained. Moreover, Germany has seen a rapid increase in the proportion of VRE among *E. faecium*, from 1.4% in 2001 to 14.5% in 2013 and then 23.8% in 2018 [30]. The cause of this is still unknown. Probably because of the stringent infection prevention and outbreak control in the Netherlands, the proportion of VRE from blood cultures among *E. faecium* never exceeded 1.5% in the Netherlands [30].

The difference in MDRO prevalence between NL-BR and DE-BR was also observed for Gram-negative MDROs. Since the Netherlands and Germany have different guidelines to classify Gram-negative bacteria as MDRO (BRMO vs 3MRGN/4MRGN) but both phenotypically test for third-generation cephalosporins, a comparison was made based on 3GCRE. The 3GCRE carriage prevalence in the DE-BR was almost twice as high (6.6%) as in the NL-BR (3.6%), but both were still lower than national averages. In 2018, the ECDC reported proportions of 3GCRE among *E. coli* and *K. pneumoniae* from blood as *E. coli* (12.2%) and *K. pneumoniae* (12.9%) for Germany and *E. coli* (7.3%) and *K. pneumoniae* (11.1%) for the Netherlands. The same year the NHIs reported a slightly lower prevalence of *E. coli* (10.7%) and *K. pneumoniae* (12.0%) in Germany and *E. coli* (6.6%) and *K. pneumoniae* (10.1%) in the Netherlands [28-30]. This highlights that important differences can be identified when studying carriage in specified populations vs looking at the proportion of invasive isolates, but that the lower carriage of Gram-negative MDROs in the participating NL-DE-BR hospitals shows the value of a regional versus a national view. Notably, in the present study, only four CRE isolates were identified, all from the DE-BR. Interestingly, when applying the country-specific guidelines to the Gram-negative MDROs study isolates, the Dutch BRMO guideline yields more MDRO than the German 3MRGN/4MRGN guideline (overall BRMO: 5.6% vs overall 3MRGN/4MRGN: 2.9%/0.1%). This difference is comparable to results from a previous study where the same guidelines were compared between the two countries [13]. Since the Dutch guideline classifies all third-generation cephalosporin-resistant *E. coli* and *Klebsiella* spp. as BRMO, while the German guideline only classifies them as MRGN if they are additionally ciprofloxacin-resistant, a higher prevalence of BRMO than MRGN was expected.

As both university and non-university hospitals participated in the study, a comparison of MDRO carriage prevalence on ICUs based on the type of hospital could be performed. In the NL-BR, no significant difference for all investigated MDROs between university and non-university hospitals was observed. In the DE-BR, on the other hand, significant differences were observed for 3GCRE, 3MRGN and BRMO between university and non-university hospitals, but not for MRSA, VRE, 4MRGN and CRE. Non-university hospitals presented a significantly higher MDRO prevalence for 3GCRE, 3MRGN and BRMO at ICU admission. Explaining this observed dissimilarity requires additional studies on e.g. hospital activity, size, staff availability, hospital geography and inter-hospital distance. A recent report highlighted that a higher density of inpatient care, a higher number of hospitals, a longer length of stay and lower staffing ratios all might facilitate MDRO dissemination [31]. Interestingly, when comparing the hospital types between the two border regions, the university hospitals had a very similar prevalence of all MDROs on ICUs. Our results show that ICUs in non-university hospitals in the DE-BR are being challenged more frequently with Gram-negative MDROs compared with MRSA and VRE. This problem seems very prominent, particularly with respect to third-generation cephalosporin resistance. This contradicts the general consensus that MDROs are less prevalent in smaller hospitals. The reason for this difference and problem is unknown and requires further investigation. However, Harbarth et al. claim that, especially in smaller hospital settings, up to one third of all hospital-associated infections can be prevented by solely improving infection prevention [32]. To investigate this, more information about the staff and patients admitted to ICUs would be required, e.g. number of staff as well as hours dedicated for infection prevention, information on severity of disease, antibiotic exposure or length of hospital stay before ICU admission.

The limitations of this study exemplify the challenge to compare AMR prevalence rates within or between healthcare regions, especially when comprising a national border. Firstly, the median screening compliance was unsatisfactory in both border regions, although significantly higher in the DE-BR (62.6%) than in the NL-BR (56.9%). Only two of 23 hospitals were equipped with sufficient staff, one on each side of the border; their screening compliance was 99.3% and 83.2%, respectively. This underlines the need for more guidance, i.e. research, and/or more staffing, education and material to implement better infection prevention and control. It also accentuates the inherently limited maximum compliance to be gained from routine wards and workflows, which is also an important point of consideration when using (inter)nationally published results. Secondly, collection of information about infection control staff, MDRO outbreaks, infections, antibiotic use and risk factors of patients was outside the scope of this study. Although this would have allowed for the analysis of origin and source of the identified MRDOs, this information was practically impossible to retrieve from the 23 different hospitals and 3,365 patients included in this study due to legislative and organisational constraints. Thirdly, the participating laboratories in this study were not homogeneous in their diagnostic test methodologies and since for most of the laboratory’s molecular confirmation, e.g. of resistance encoding genes, was not part of their standard operating procedures, it was also not included in the study protocol. Fourthly, not all hospitals conducted the screening in the same 8 consecutive weeks, as this was practically unfeasible. While this might have improved comparability, others found almost no seasonality in bacterial bloodstream infections and we therefore consider this issue to be of low impact [33].

This study highlights the importance of a regional and cross-border approach in any European cross-border region to illustrate the difference of AMR prevalence between the regions and to highlight potential differences with country-wide reports. Moreover, the focus on routine workflows in both the hospital and laboratories make this study valuable, since it offers an honest perspective on the reality. To be able to emphasise on this further, attaining a deeper level of detail is a prerequisite, e.g. by collecting information about staff on the wards and infection control staff, MDRO outbreaks, infections, antibiotic use and risk factors of patients. Standard reporting based on the Nomenclature of Territorial Units for Statistics (NUTS) on a NUTS3 or at least NUTS2 level instead of NUTS1 or the national level would also improve the resolution of the AMR prevalence within a country or healthcare region and improve the understanding thereof. Interestingly, comparisons with national data on MDRO proportions as reported by the ECDC and the respective NHIs revealed rather low numbers of submitted isolates, which highlights a bottleneck of using this data source. Moreover, only a limited number of hospitals, mostly large (university) hospitals especially in Germany, actively participate in national or international surveillance systems arguing for the inclusion of small and medium-sized hospitals when determining and analysing MDRO prevalences. Additionally, generalising guidelines and definitions between countries, preferably on the European level, will improve comparability between countries which is of great importance for cross-border regions. 

In conclusion, healthcare systems, geographic nature and guidelines are very different between the two countries, although the prevalence of MDROs do not reflect these differences. This indicates that MDROs are not contained by political borders and should be treated as a non-country specific problem. Proportions of MDROs of certain pathogens, as reported on the national and international level, do not reflect MDRO prevalence in the patient or general population. This should be taken into consideration when interpreting reports on the country or even continental level.

## Supplementary Data

806000 Supplement

## Supplementary Data

46000 Supplementary Table 2
