# Supplementary material for: A prospective multicentre screening study on multidrug-resistant organisms in intensive care units in the Dutch–German cross-border region, 2017 to 2018: the importance of healthcare structures
Source: Euro Surveill. 2022 Feb 3;27(5):2001660. doi: 10.2807/1560-7917.ES.2022.27.5.2001660 (PMC8815100; doi:10.2807/1560-7917.ES.2022.27.5.2001660)
Supplement: Supplement [file 20-01660_GLASNER_Supplement.pdf]

This supplementary material is hosted by Eurosurveillance as supporting information alongside the article "A Prospective Multicentre MDRO Screening Study on ICU's in the Dutch-German Cross-Border Region (2017-2018): the Importance of Healthcare Structures", on behalf of the authors, who remain responsible for the accuracy and appropriateness of the content. The same standards for ethics, copyright, attributions and permissions as for the article apply. Supplements are not edited by Eurosurveillance and the journal is not responsible for the maintenance of any links or email addresses provided therein.

Supplementary Table S1

Laboratory name and place	Hospital name and place	Sample location	Type of enrichment broth	Type of selective agar plate
Certe, Groningen	Martini, Groningen	Nasopharyngeal	selective	ChromID MRSA (bioMérieux)
Certe, Groningen	Martini, Groningen	Rectum	non-selective	ChromID ESBL (bioMérieux)
Certe, Groningen	Martini, Groningen	Rectum	selective	ChromID VRE (bioMérieux)
Certe, Groningen	Scheper, Emmen	Nasopharyngeal	selective	ChromID MRSA (bioMérieux)
Certe, Groningen	Scheper, Emmen	Rectum	non-selective	ChromID ESBL (bioMérieux)
Certe, Groningen	Scheper, Emmen	Rectum	selective	ChromID VRE (bioMérieux)
Christophorus, Coesfeld/Dülmen	Christophorus-Kliniken, Coesfeld/Dülmen	Nasopharyngeal	none	MRSA Brilliance 2 Agar (Oxoid)
Christophorus, Coesfeld/Dülmen	Christophorus-Kliniken, Coesfeld/Dülmen	Rectum	none	VRE agar (bioMérieux)
Christophorus, Coesfeld/Dülmen	Christophorus-Kliniken, Coesfeld/Dülmen	Rectum	none	BD BBL CHROMagar ESBL
CWZ, Nijmegen	CWZ, Nijmegen	Nasopharyngeal	none	MRSA agar (bioMérieux)
CWZ, Nijmegen	CWZ, Nijmegen	Rectum	none	ESBL agar (bioMérieux)
CWZ, Nijmegen	CWZ, Nijmegen	Rectum	none	Brilliance VRE agar (Oxoid)
IMHL, Lippe	Klinikum Lippe, Detmold	Nasopharyngeal	HHB	BRILLIANCE MRSA 2
IMHL, Lippe	Klinikum Lippe, Detmold	Rectum	none	chromID VRE bioMérieux
IMHL, Lippe	Klinikum Lippe, Detmold	Rectum	none	BD MacConkey II Agar + CTX
IMHL, Lippe	Klinikum Lippe, Detmold	Rectum	none	ESBL bioMérieux
IMHL, Lippe	Klinikum Lippe, Detmold	Rectum	none	Brilliance ESBL AGAR Oxoid Microbiology Products
Isala, Zwolle	Isala, Zwolle	Nasopharyngeal	none	CLED; blood agar with aztreonam & colistin
Isala, Zwolle	Isala, Zwolle	Rectum	none	CLED; blood agar with aztreonam & colistin
Izore, Leeuwarden	MCL, Leeuwarden	Nasopharyngeal	selective	MRSA agar (Oxoid); blood agar plate
Izore, Leeuwarden	MCL, Leeuwarden	Rectum	selective	EBSA agar (Tritium)
Izore, Leeuwarden	MCL, Leeuwarden	Rectum	selective	Primary: PCR on vanA/vanB, Secondary: Brilliance VRE Agar (Oxoid)
Izore, Leeuwarden	Tjongerschans, Heerenveen	Nasopharyngeal	selective	MRSA agar (Oxoid); blood agar plate
Izore, Leeuwarden	Tjongerschans, Heerenveen	Rectum	selective	EBSA agar (Tritium)
Izore, Leeuwarden	Tjongerschans, Heerenveen	Rectum	selective	Primary: PCR on vanA/vanB, Secondary: Brilliance VRE Agar (Oxoid)

LabMicTA, Enschede	MST, Enschede	Nasopharyngeal	selective	MRSA2 Brilliance screening agar (Oxoid) (Direct)
LabMicTA, Enschede	MST, Enschede	Rectum	non-selective	BRMO-1 : ETP-CLOX (MediaProducts)
LabMicTA, Enschede	MST, Enschede	Rectum	selective	B-VRE agar (Oxoid)
Laborarztpraxis Osnabrück	Ammerland Klinik, Westerstede	Nasopharyngeal	non-selective	Chrom ID MRSA Smart (bioMérieux)
Laborarztpraxis Osnabrück	Ammerland Klinik, Westerstede	Rectum	non-selective	Chrom ID VRE (bioMérieux)
Laborarztpraxis Osnabrück	Ammerland Klinik, Westerstede	Rectum	non-selective	Brilliance ESBL (Oxoid)
Laborarztpraxis Osnabrück	Bonifatius-Hospital, Lingen	Rectum	non-selective	Brilliance ESBL (Oxoid)
Laborarztpraxis Osnabrück	Bonifatius-Hospital, Lingen	Nasopharyngeal	non-selective	Chrom ID MRSA Smart (bioMérieux)
Laborarztpraxis Osnabrück	Bonifatius-Hospital, Lingen	Rectum	non-selective	Chrom ID VRE (bioMérieux)
Laborarztpraxis Osnabrück	Borromäus-Hospital, Leer	Nasopharyngeal	non-selective	Chrom ID MRSA Smart (bioMérieux)
Laborarztpraxis Osnabrück	Borromäus-Hospital, Leer	Rectum	non-selective	Chrom ID VRE (bioMérieux)
Laborarztpraxis Osnabrück	Borromäus-Hospital, Leer	Rectum	non-selective	Brilliance ESBL (Oxoid)
Laborarztpraxis Osnabrück	Bundeswehrkrankenhaus, Westerstede	Nasopharyngeal	non-selective	Chrom ID MRSA Smart (bioMérieux)
Laborarztpraxis Osnabrück	Bundeswehrkrankenhaus, Westerstede	Rectum	non-selective	Chrom ID VRE (bioMérieux)
Laborarztpraxis Osnabrück	Bundeswehrkrankenhaus, Westerstede	Rectum	non-selective	Brilliance ESBL (Oxoid)
Laborarztpraxis Osnabrück	CKQ, Quakenbrück	Nasopharyngeal	non-selective	Chrom ID MRSA Smart (bioMérieux)
Laborarztpraxis Osnabrück	CKQ, Quakenbrück	Rectum	non-selective	Chrom ID VRE (bioMérieux)
Laborarztpraxis Osnabrück	CKQ, Quakenbrück	Rectum	non-selective	Brilliance ESBL (Oxoid)
Laborarztpraxis Osnabrück	Elisabeth-Krankenhaus, Thuine	Nasopharyngeal	non-selective	Chrom ID MRSA Smart (bioMérieux)
Laborarztpraxis Osnabrück	Elisabeth-Krankenhaus, Thuine	Rectum	non-selective	Chrom ID VRE (bioMérieux)
Laborarztpraxis Osnabrück	Elisabeth-Krankenhaus, Thuine	Rectum	non-selective	Brilliance ESBL (Oxoid)
Laborarztpraxis Osnabrück	Euregio-Klinik, Nordhorn	Nasopharyngeal	non-selective	Chrom ID MRSA Smart (bioMérieux)
Laborarztpraxis Osnabrück	Euregio-Klinik, Nordhorn	Rectum	non-selective	Chrom ID VRE (bioMérieux)
Laborarztpraxis Osnabrück	Euregio-Klinik, Nordhorn	Rectum	non-selective	Brilliance ESBL (Oxoid)
Laborarztpraxis Osnabrück	Klinikum-Osnabrück, Osnabrück	Nasopharyngeal	non-selective	Chrom ID MRSA Smart (bioMérieux)
Laborarztpraxis Osnabrück	Klinikum-Osnabrück, Osnabrück	Rectum	non-selective	Chrom ID VRE (bioMérieux)
Laborarztpraxis Osnabrück	Klinikum-Osnabrück, Osnabrück	Rectum	non-selective	Brilliance ESBL (Oxoid)
Laborarztpraxis Osnabrück	Paracelsus-Klinik, Osnabrück	Nasopharyngeal	non-selective	Chrom ID MRSA Smart (bioMérieux)
Laborarztpraxis Osnabrück	Paracelsus-Klinik, Osnabrück	Rectum	non-selective	Chrom ID VRE (bioMérieux)
Laborarztpraxis Osnabrück	Paracelsus-Klinik, Osnabrück	Rectum	non-selective	Brilliance ESBL (Oxoid)
Laborarztpraxis Osnabrück	Schüchtermann-Klinik, Bad Rothenfelde	Nasopharyngeal	non-selective	Chrom ID MRSA Smart (bioMérieux)
Laborarztpraxis Osnabrück	Schüchtermann-Klinik, Bad Rothenfelde	Rectum	non-selective	Chrom ID VRE (bioMérieux)
Laborarztpraxis Osnabrück	Schüchtermann-Klinik, Bad Rothenfelde	Rectum	non-selective	Brilliance ESBL (Oxoid)
Laborarztpraxis Osnabrück	St. Josef-Hospital, Cloppenburg	Nasopharyngeal	non-selective	Chrom ID MRSA Smart (bioMérieux)
Laborarztpraxis Osnabrück	St. Josef-Hospital, Cloppenburg	Rectum	non-selective	Chrom ID VRE (bioMérieux)
Laborarztpraxis Osnabrück	St. Josef-Hospital, Cloppenburg	Rectum	non-selective	Brilliance ESBL (Oxoid)
Laborarztpraxis Osnabrück	St. Marien-Hospital, Vechta	Nasopharyngeal	non-selective	Chrom ID MRSA Smart (bioMérieux)
Laborarztpraxis Osnabrück	St. Marien-Hospital, Vechta	Rectum	non-selective	Chrom ID VRE (bioMérieux)
Laborarztpraxis Osnabrück	St. Marien-Hospital, Vechta	Rectum	non-selective	Brilliance ESBL (Oxoid)
UKM, Münster	UKM, Münster	Nasopharyngeal	non-selective	chromID MRSA (bioMérieux)
UKM, Münster	UKM, Münster	Rectum	none	VRESelect (Biorad)

UKM, Münster	UKM, Münster	Rectum	none	chromID ESBL (bioMérieux)
UMCG, Groningen	UMCG, Groningen	Nasopharyngeal	non-selective	ChromID MRSA Agar (bioMérieux) & Aztreonam Blood Agar (Mediaproducts)
UMCG, Groningen	UMCG, Groningen	Rectum	selective	Primary: PCR on vanA/vanB, Secondary: Brilliance VRE Agar (Oxoid)
UMCG, Groningen	UMCG, Groningen	Rectum	none	3 Selective Agar Plates (Mediaproducts)

Supplementary Figure S1

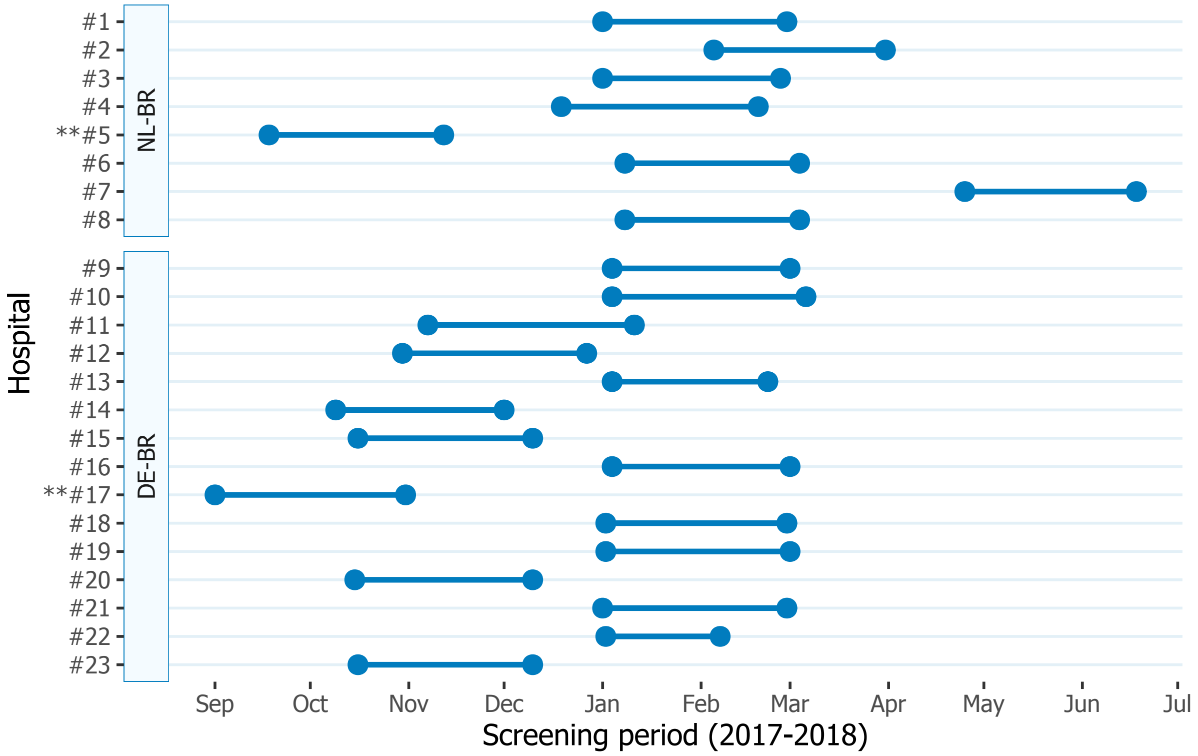

Supplementary Figure 1. Screening period per hospital. All hospitals screened between September 2017 and July 2018. Hospitals #5 and #17 were university hospitals and started almost immediately after the start of the study. Hospital #7 could only start in May 2018 due to lack of available personnel.

Supplementary Material

This supplementary material is hosted by Eurosurveillance as supporting information alongside the article "A Prospective Multicentre MDRO Screening Study on ICU's in the Dutch-German Cross-Border Region (2017-2018): the Importance of Healthcare Structures", on behalf of the authors, who remain responsible for the accuracy and appropriateness of the content. The same standards for ethics, copyright, attributions and permissions as for the article apply. Supplements are not edited by Eurosurveillance and the journal is not responsible for the maintenance of any links or email addresses provided therein.

Overview and summary of MDRO definitions based on different national and international guidelines mentioned and used in the manuscript "A Prospective Multicentre MDRO Screening Study on ICU's in the Dutch-German Cross-Border Region (2017-2018): the Importance of Healthcare Structures".

Robert Koch-Institut, Germany: Empfehlung der Kommission für Krankenhaushygiene und Infektionsprävention (KRINKO) beim Robert Koch-Institut (RKI)

The original German version:

Antibiotikagruppe	Leitsubstanz	Enterobacterales		*Pseudomonas aeruginosa*		*Acinetobacter baumannii*	
		3MRGN[1]	4MRGN[2]	3MRGN[1]	4MRGN[2]	3MRGN[1]	4MRGN[2]
Acylureidopenicilline	Piperacillin	R	R	Nur eine der 4 Antibiotikagruppen wirksam (S oder I)	R	R	R
3./4. Generations-Cephalosporine	Cefotaxim und/ oder Ceftazidim	R	R		R	R	R
Carbapeneme	Imipenem und/ oder Meropenem	S oder I	R		R	S oder I	R
Fluorchinolone	Ciprofloxacin	R	R		R	R	R
			oder Nachweis einer Carbapenemase[3]		oder Nachweis einer Carbapenemase[3]		oder Nachweis einer Carbapenemase[3]

Tab. 2: Neue Klassifizierung multiresistenter gramnegativer Stäbchen auf Basis ihrer phänotypischen Resistenzeigenschaften bei Anwendung des EUCAST-Systems

(R = resistent, I = sensibel bei erhöhter *(Increased)* Dosierung/Exposition, S = sensibel bei normaler Dosierung)

1 3MRGN (**M**ulti**r**esistente **g**ram**n**egative Stäbchen mit Resistenz gegen **3** der 4 Antibiotikagruppen)

2 4MRGN (**M**ulti**r**esistente **g**ram**n**egative Stäbchen mit Resistenz gegen **4** der 4 Antibiotikagruppen)

3 Unabhängig vom Ergebnis der phänotypischen Resistenzbestimmung für Carbapeneme sowie der anderen drei Substanzklassen

The translated English version:

<table>
<tr><th>Antibiotic Group</th><th>Main component</th><th colspan="2">Enterobacterales</th><th colspan="2">Pseudomonas aeruginosa</th><th colspan="2">Acinetobacter baumannii</th></tr>
<tr><td></td><td></td><td>3MRGN[1]</td><td>4MRGN[2]</td><td>3MRGN[1]</td><td>4MRGN[2]</td><td>3MRGN[1]</td><td>4MRGN[2]</td></tr>
<tr><td>Acyl-ureidopenicillins</td><td>Piperacillin</td><td>R</td><td>R</td><td rowspan="4">Only one out of four antibiotic groups effective (suscep-tible)</td><td>R</td><td>R</td><td>R</td></tr>
<tr><td>3rd/4th gen. cephalosporins</td><td>Cefotaxime and/or ceftazidime</td><td>R</td><td>R</td><td>R</td><td>R</td><td>R</td></tr>
<tr><td>Carbapenems</td><td>Imipenem and/or meropenem</td><td>S or I</td><td>R</td><td>R</td><td>S or I</td><td>R</td></tr>
<tr><td>Fluoroquinolones</td><td>Ciprofloxacin</td><td>R</td><td>R</td><td>R</td><td>R</td><td>R</td></tr>
<tr><td></td><td></td><td></td><td>or the detection of a carbapenemase [3]</td><td></td><td>or the detection of a carbapenemase [3]</td><td></td><td>or the detection of a carbapenemase [3]</td></tr>
</table>

Table 1. Classification of multiresistant gram-negative rods based on their phenotypic resistance properties from "Hygienic measures in case of infections or colonisation with multiresistant gram-negative rods" (Bundesgesundheitsblatt 10/2012).
(R = resistant or intermediate susceptible, S = susceptible).
[1] 3MRGN (multiresistant Gram-negative rods with resistance to 3 of the 4 antibiotic groups)
[2] 4MRGN (multidrug-resistant Gram-negative rods with resistance to 4 of the 4 antibiotic groups)
[3] All carbapenem-resistant isolates are considered 4MRGN irrespectively of the test results for fluoroquinolones.

Information was extracted from the following links on the 23rd of February 2021:
https://edoc.rki.de/bitstream/handle/176904/253/21obND4dxM.pdf?sequence=1&isAllowed=y
https://www.rki.de/DE/Content/Infekt/EpidBull/Archiv/2019/Ausgaben/09_19.pdf?__blob=publicationFile

‘Werkgroep Infectiepreventie’ (WIP) in the Netherlands

The original Dutch version:

BRMO (Bijzonder resistente micro-organismen): (pathogene) micro-organismen die ongevoelig zijn voor de meest geëigende (dus eerste keus) antibiotica of tegen een combinatie van therapeutisch belangrijke antibiotica en die zonder aanvullende maatregelen tot verspreiding kunnen leiden.

Toelichting bij de Tabellen 1-3:

A. Resistentie;

B. Combinatie van resistentie voor antibiotica uit tenminste twee van de aangeduide antibioticagroepen of middelen;

C. Combinatie van resistentie voor antibiotica uit tenminste drie van de aangeduide antibioticagroepen of middelen.

Tabel 1. Resistentiecriteria voor Enterobacteriaceae

Gram-negatieve staven	ESBL	Fluor-chinolonen	Amino-glycosiden	Carbapenemase positief
Enterobacteriaceae*	A	B	B	A

*Ondanks het feit dat uitbraken met plasmidaal AmpC producerende Enterobacteriaceae in de literatuur zijn beschreven is dit resistentiemechanisme niet opgenomen in Tabel 1 (7-9). In Nederland zijn dergelijke uitbraken nog niet waargenomen en is de prevalentie van plasmidaal AmpC resistentie laag (ongepubliceerde gegevens Voets et al. 2009: 5% resp. 0% onder 3e generatie cephalosporine resistente *E. coli* and *K. pneumoniae*). Bovendien zijn fenotypische methoden voor het vaststellen van plasmidale AmpC resistentie nog niet gevalideerd voor routinematige doeleinden en kunnen de in de literatuur voorgestelde experimentele methoden niet goed onderscheid maken tussen chromosomale en plasmidale AmpC resistentie (7-10).
Ook voor uitbraken met *Enterobacteriaceae* met plasmidale combinatieresistentie voor fluorchinolonen en aminoglycosiden (aac(6’)-Ib-cr, qnrA, qnrB, qnrC, qnrS, qepA) geldt dat deze in Nederland niet tot nauwelijks zijn waargenomen. Ook hierbij zijn fenotypische methoden niet geschikt om onderscheid te maken tussen chromosomale en plasmidale resistentie.

The translated English version:

BRMO (Particularly Resistant Microorganisms, “Bijzonder resistente micro-organismen”): (pathogenic) microorganisms that are resistant to the most appropriate (i.e. first choice) antibiotics or to a combination of therapeutically important antibiotics and that can lead to spreading without additional measures.

Note to Tables 1-3:

A. Resistance;
B. Combination of resistance for antibiotics from at least two of the mentioned antibiotic groups;
C. Combination of resistance for antibiotics from at least three of the mentioned antibiotic groups.

Table 1. Resistance criteria for *Enterobacteriaceae*.

Gram-negative rods	ESBL	Fluoroquinolones	Aminoglycosides	Carbapenemase-positive
Enterobacteriaceae	A	B	B	A

*Despite the fact that outbreaks with plasmidally AmpC-producing Enterobacteriaceae have been described in the literature, this resistance mechanism is not included in Table 1 [7-9]. In the Netherlands, such outbreaks have not yet been observed and the prevalence of plasmid AmpC resistance is low (unpublished data Voets et al. 2009: 5% and 0%, respectively, among 3rd generation cephalosporin-resistant *E. coli* and *K. pneumoniae*). Moreover, phenotypic methods for determining plasmidal AmpC resistance have not yet been validated for routine purposes and the experimental methods proposed in the literature cannot properly distinguish between chromosomal and plasmidal AmpC resistance [7-10].
Also for outbreaks with Enterobacteriaceae with plasmidal combination resistance to fluoroquinolones and aminoglycosides (aac(6')-Ib-cr, qnrA, qnrB, qnrC, qnrS, qepA) applies that these have not or hardly been observed in the Netherlands. Again, phenotypic methods are not suitable to distinguish between chromosomal and plasmidal resistance.

Information was extracted from the following links on the 23rd of February 2021:
https://www.rivm.nl/documenten/wip-richtlijn-brmo
https://lci.rivm.nl/richtlijnen/brmo

European Centre for Disease Prevention and Control (ECDC)

EARS-Net performs surveillance of antimicrobial susceptibility of seven bacterial pathogens commonly causing infections in humans:

Escherichia coli
Klebsiella pneumoniae
Pseudomonas aeruginosa
Acinetobacter species
Streptococcus pneumoniae
Staphylococcus aureus
Enterococcus faecalis
Enterococcus faecium

The EARS-Net reporting protocol defines the panels of antimicrobial agent combinations under surveillance for each species. In addition, the EUCAST guidelines for the detection of resistance mechanisms and specific types of resistance of clinical and/or epidemiological importance explain the mechanisms of resistance and describe the recommended methods of detection for key species–antimicrobial group combinations. For further details on EARS-Net data collection and analysis, please refer to the latest EARS-Net reporting protocol.

Surveillance Atlas based on species and the selection of:

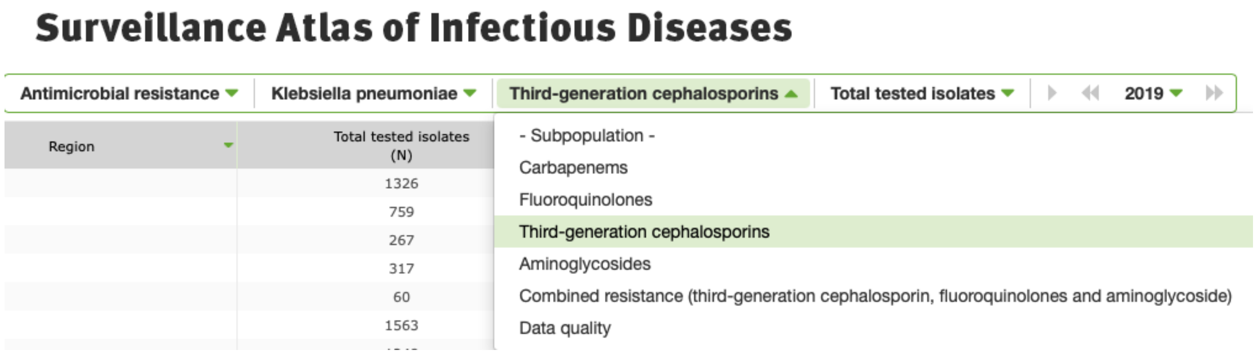

Information was extracted from the following links on the 23rd of February 2021:
https://www.ecdc.europa.eu/sites/default/files/documents/surveillance-antimicrobial-resistance-Europe-2019.pdf
https://www.ecdc.europa.eu/en/about-us/networks/disease-networks-and-laboratory-networks/ears-net-data
https://atlas.ecdc.europa.eu/public/index.aspx?Dataset=27&HealthTopic=4

World Health Organization (WHO)

WHO priority pathogens list for R&D of new antibiotics:

Priority 1: CRITICAL
Acinetobacter baumannii, carbapenem-resistant
Pseudomonas aeruginosa, carbapenem-resistant
Enterobacteriaceae, carbapenem-resistant, ESBL-producing

Priority 2: HIGH
Enterococcus faecium, vancomycin-resistant
Staphylococcus aureus, methicillin-resistant, vancomycin-intermediate and resistant
Helicobacter pylori, clarithromycin-resistant
Campylobacter spp., fluoroquinolone-resistant
Salmonellae, fluoroquinolone-resistant
Neisseria gonorrhoeae, cephalosporin-resistant, fluoroquinolone-resistant

Priority 3: MEDIUM
Streptococcus pneumoniae, penicillin-non-susceptible
*Haemophilus influ*enzae, ampicillin-resistant
Shigella spp., fluoroquinolone-resistant

Information was extracted from the following link on the 23rd of February 2021:
https://www.who.int/news/item/27-02-2017-who-publishes-list-of-bacteria-for-which-new-antibiotics-are-urgently-needed
